# Intelligent Diagnosis and Classification of Keratitis

**DOI:** 10.3390/diagnostics12061344

**Published:** 2022-05-28

**Authors:** Hiam Alquran, Yazan Al-Issa, Mohammed Alsalatie, Wan Azani Mustafa, Isam Abu Qasmieh, Ala’a Zyout

**Affiliations:** 1 Department of Biomedical Engineering, Jordan University of Science and Technology, Irbid 22110, Jordan; heyam.q@yu.edu.jo; 2 Biomedical Systems and Medical Informatics Engineering, Yarmouk University, Irbid 21163, Jordan; iabuqasmieh@yu.edu.jo (I.A.Q.); alzuet@yu.edu.jo (A.Z.); 3 Department of Computer Engineering, Yarmouk University, Irbid 21163, Jordan; alissay@yu.edu.jo; 4 The Institute of Biomedical Technology, King Hussein Medical Center, Royal Jordanian Medical Service, Amman 11855, Jordan; mhmdsliti312@gmail.com; 5 Faculty of Electrical Engineering Technology, Campus Pauh Putra, Universiti Malaysia Perlis, Arau 02600, Perlis, Malaysia; 6 Advanced Computing (AdvComp), Centre of Excellence (CoE), Campus Pauh Putra, Universiti Malaysia Perlis (UniMAP), Arau 02600, Perlis, Malaysia

**Keywords:** corneal ulcer, deep learning, ResNet101, PCA

## Abstract

A corneal ulcer is an open sore that forms on the cornea; it is usually caused by an infection or injury and can result in ocular morbidity. Early detection and discrimination between different ulcer diseases reduces the chances of visual disability. Traditional clinical methods that use slit-lamp images can be tiresome, expensive, and time-consuming. Instead, this paper proposes a deep learning approach to diagnose corneal ulcers, enabling better, improved treatment. This paper suggests two modes to classify corneal images using manual and automatic deep learning feature extraction. Different dimensionality reduction techniques are utilized to uncover the most significant features that give the best results. Experimental results show that manual and automatic feature extraction techniques succeeded in discriminating ulcers from a general grading perspective, with ~93% accuracy using the 30 most significant features extracted using various dimensionality reduction techniques. On the other hand, automatic deep learning feature extraction discriminated severity grading with a higher accuracy than type grading regardless of the number of features used. To the best of our knowledge, this is the first report to ever attempt to distinguish corneal ulcers based on their grade grading, type grading, ulcer shape, and distribution. Identifying corneal ulcers at an early stage is a preventive measure that reduces aggravation and helps track the efficacy of adapted medical treatment, improving the general public health in remote, underserved areas.

## 1. Introduction

Corneal ulcer (CU), also known as keratitis, is an infection or inflammation that affects the transparent anterior portion of the eye that covers the iris, which is known as the cornea [1]. Corneal ulcer is a major cause of sight loss and might be responsible for 1.5–2.0 million blindness cases every year [2]. The source of corneal ulcer can be viral, bacterial, fungal, or parasitic. The symptoms include pain, ache, redness, blurry vision, and sensitivity to bright light. Traditional methods that use slit-lamp images and slit-lamp microscopy for diagnosing corneal ulcers are subjective and time-consuming, and they are highly dependent on the ophthalmologist expertise. It is a preventable and treatable disease, as early and timely recognition of corneal ulcer can stop the deterioration and help maintain a patient’s visual integrity.

Developments in staining techniques help investigators numerically detect and diagnose ulcers. Fluorescein is a widely used dye in ophthalmology for the diagnosis and evaluation of the corneal integrity and the optical exterior. Many ophthalmologists use fluorescein corneal staining technique together with slit-lamp microscopy to successfully diagnose and analyze corneal ulcers. Several methods for corneal ulcer segmentation and classification are found in the literature [3,4,5,6,7]. Both automatic and manual segmentation techniques help identify the severity of the ulcer. Automatic and semiautomatic segmentation algorithms that use artificial intelligence require large training datasets [8], whereas manual segmentation is a highly subjective and time-consuming technique. Finally, the region containing the ulcer is distinguished by applying a glowing green dye against the rest of the cornea, which appears brown or blue [8,9,10,11].

The aim of this study is to propose an automated system to distinguish different corneal ulcers types and to compare hand and automatic features extraction using the publicly available SUSTech-SYSU dataset. The suggested approach distinguishes between different corneal ulcer images using handcraft and automatic features extracted from gray-level and colored images. The colored features are extracted from three color spaces: red green blue (RGB), luminance chroma-blue chroma-red (YCbCr), and hue saturation value (HSV). In addition, this work analyzed the performance of various feature selection methods, such as principal component analysis (PCA), infinite latent feature selection (ILFS) [12], ensemble-based classifier feature selection (ECFS) [13], and Fisher [13], to determine whether a comparable performance can be obtained with fewer features. To the best of our knowledge, this is the first study to ever attempt to distinguish corneal ulcers based on their type grading, grade grading, and general pattern (ulcer shape and distribution) using fluorescein staining images. Early and timely diagnosis of corneal ulcers can help provide clinically adapted therapy and can assist track the therapeutic treatment efficiently.

## 2. Literature Review

In this section, we examine the most pertinent literature that has attempted to categorize different types of corneal ulcers to prevent corneal blindness. Ashrafi Akram and Rameswar Debnath proposed an automated system to detect the presence or absence of corneal ulcer disease from a facial image taken by a digital camera. The eye part of the face is segmented using Haar cascade classifiers, and the iris and sclera regions were segmented by applying Hough gradient and active contour techniques to localize the ulcer area. The model achieved an accuracy of 99.43%, a sensitivity of 98.78%, and a specificity of 98.6% [3]. Zhongwen Li et al. used three pretrained models, particularly DenseNet121, ResNet50, and Inception, to discriminate between normal cornea, keratitis, and other abnormalities. The models were trained using 6567 slit-lamp images from different sources, and DenseNet121 gave the best results and achieved an AUC > 0.96 [4].

Multiple researches were conducted using the SUSTech-SYSU dataset, which consists of 712 fluorescein staining images to differentiate various corneal ulcers. In 2020, a modified VGG network was proposed by Ningbiao Tang et al. for automatic classification of corneal ulcers. The framework had fewer parameters and better performance compared with the traditional convolutional neural network (CNN). It discriminated between point-like, flaky, and point-flaky mixed ulcers. The performance of the modified architecture exceeded that of VGG16 and AlexNet, as it achieved 88.89% accuracy, 71.93% sensitivity, and 71.39% F1-score [5]. In 2020, Zhonghua Wang et al. suggested two binary models to classify three types of corneal ulcers. The first model discriminates point-like against mixed and flaky ulcers, and the second model distinguishes between mixed and flaky ulcers. The proposed pipeline achieved an accuracy of 85.8% [6].

In 2021, Kasemsit Teeyapan et al. trained 15 different convolutional neural networks (CNNs) using the SUSTech-SYSU dataset to discriminate between early and advanced stages of corneal ulcer. The best results were obtained using ResNet50 with a 95.1% accuracy, 94.37% sensitivity, and F1-score of 95.04% [7]. In 2021, Jan Gross et al. used transfer learning to compare the performance of VGG16, VGG19, Xception, and ResNet50 pretrained models in distinguishing between different corneal ulcers. VGG16 discriminated between general ulcer patterns with 92.73% accuracy. Images were preprocessed using thresholding and data augmentation, and the proposed method avoids errors resulting from light reflection during diagnostic imaging [14].

## 3. Materials and Methods

**Figure 1:**
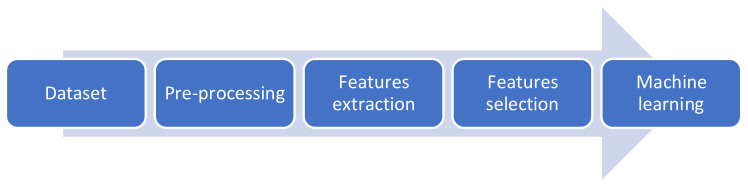
Flow chart of the proposed methodology.

Figure 1 illustrates the flowchart of the multistage method employed in this paper. First, the SUSTech-SYSU dataset was resized and preprocessed, and next, the features were extracted either by hand or automatically, and later, various feature selection methods were employed to uncover the most relevant features; finally, machine learning techniques were utilized to classify images into different categories. The following subsections describes each step in detail.

### 3.1. Dataset

**Figure 2:**
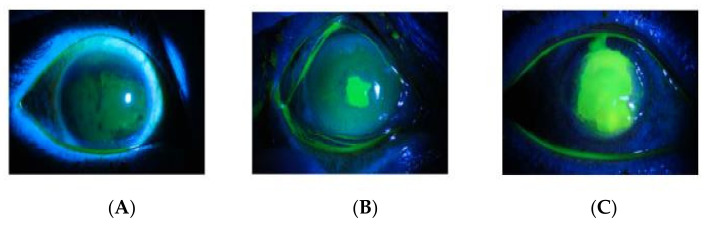
(**A**) point-like, (**B**) point-flaky, (**C**) and flaky corneal.

This study utilized the labeled corneal ulcer images from the publicly available SUSTech-SYSU database [15,16]. The dataset contains a total of 712 fluorescein-stained images that captured the ocular surfaces and were collected from patients with various corneal ulcer degrees. Images are 24-bit RGB colored with a 2592 × 1728 pixels’ spatial resolution, and each picture contained only one corneal image. In general, there are three ways to categorize the images and the details are shown in Table 1:

- General pattern: Separate the images according to the shape and distribution characteristics of the corneal ulcer. They can be classified into three categories shown in Figure 2.
- Type grading: Separate the images according to the corneal ulcer’s specific pattern. They can be classified into five categories: Type 0 (no ulcer of the corneal epithelium), Type 1 (micro punctate), Type 2 (macro punctate), Type 3 (coalescent macro punctate), and Type 4 (patch ≥ 1 mm).
- Grade grading: Separate the images according to the corneal ulcer’s severity degree (grade grading). They can be classified into four categories: Grade 0, Grade 1, Grade 2, Grade 3, and Grade 4, where Grade 0 indicates that there is no ulcer, and Grade 4 indicates that the ulcer involves the central optical zone.

Different classification problems used different datasets. The corneal ulcer’s general pattern used a dataset consisting of 802 images after augmentation, 712 images were used in Model 1, and 381 images were used in Model 2. The corneal ulcer’s specific pattern (type grading) used a dataset consisting of 2179 images after augmentation. Finally, for the corneal ulcer’s severity degree (grade grading) problem, a dataset consisting of 1239 images after augmentation was used. All datasets were divided into training and testing sets, each consisting of 70% and 30% of the dataset, respectively.

**Figure 3:**
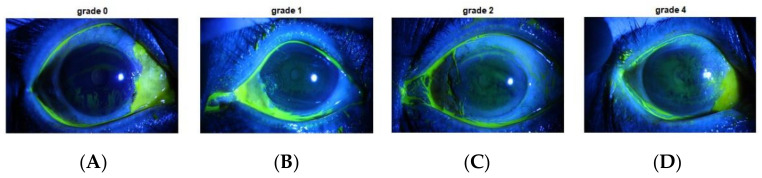
(**A**) Grade 0, (**B**) Grade 1, (**C**) Grade 2, and (**D**) Grade 4.

Figure 3 describes the grade grading category.

**Figure 4:**
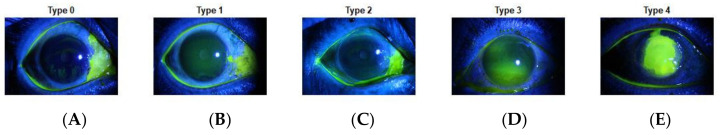
(**A**) Type 0, (**B**) Type 1, (**C**) Type 2, (**D**) Type 4, and (**E**) Type 4.

Figure 4 represents the classes of type grade.

### 3.2. Image Augmentation

The image augmentation technique enlarges the existing data to create more data for the model training process. To build a balanced dataset, several image augmentation techniques were applied, such as image rotation in different angles by 0°, 45°, 60°, 180°, and 360° degrees; isotropic scaling by factors of 0.1, 0.2, 0.5, and 0.9; and reflection in both left-right and top-bottom directions [17]. These techniques were applied on the datasets to enlarge the number of images The augmentation process considered the number of original images in each class for all three datasets. Some classes do not need augmentation because the number of images is sufficient to carry out the classification task. Therefore, no augmentation techniques were applied. On the other hand, images in other classes are not sufficient to build a robust classifier model. Therefore, various augmentation techniques were applied, and the augmentation multiplier varied between 1 to 15 based on the original number of images to expand these classes. The augmentation process is started by augmenting the original images with specific angle, scale, direction, and reflection. The resultant augmented images are then saved. After one augmentation round, if the number of images is sufficient, the resultant augmented images are then used for distinguishing between different ulcers. However, if the augmented images are still not sufficient to build a reliable model, the augmentation procedure is repeated on the original images using different augmentation parameters. The process is repeated until the data are large and appropriate enough to guarantee successful discrimination. The number of images for each class before and after augmentation in addition to the augmentation multiplier used are illustrated in Table 2, Table 3 and Table 4 for each dataset.

### 3.3. Image Preprocessing

**Figure 5:**
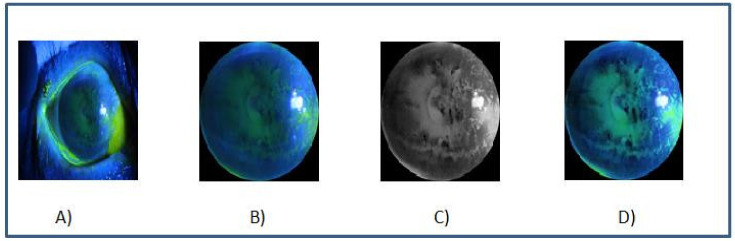
(**A**) Original image, (**B**) cornea area, (**C**) enhanced gray image, and (**D**) final enhanced image.

The purpose of the pretreatment stage is to focus on the corneal surface instead of the conjunctival areas, as it is the most commonly and highly stained area in corneal epithelial injuries [15]. To better the computational efficiency, all images were resized to a fixed input size of 256 × 256 pixels. The corneal region image is enhanced using the colorized image enhancement method. Initially, the images are converted into gray-scale level, then morphological opening operation was applied to remove the non-uniform illuminated background, and next, the contrast is adjusted using histogram equalization. Finally, the RGB image is converted to the HSV color space, and the V channel is replaced with an enhanced gray image. Figure 5 displays the output after each preprocessing stage.

### 3.4. Feature Extraction

Feature extraction captures the visual content of an image for the purpose of indexing and retrieval. An image can be expressed by a set of low-level and high-level descriptors; low-level features can be either general features or domain-specific features [18].

#### 3.4.1. Hand Crafted Feature Extraction

Textured features are extracted from gray-level images, and colored features are extracted from the three different color spaces: red green blue (RGB), luminance chroma-blue chroma-red (YCbCr), and hue saturation value (HSV). The three-color spaces are divided into nine distinct channels, namely R, G, B, H, S, V, Y, Cb, and Cr [19]. Each color space looks at the image from a different angle and provides a different way to identify features in an image.

Overall, a total of 60 features were manually extracted from the enhanced image as well as 24 textured features and 36 colored features. The 24 gray-level co-occurrence two-dimensional matrix (GLCM) features were extracted after the image was transformed to gray-level images. GLCM is a technique that allows extraction of statistical information from the image regarding the pixel distributions. It is an effective method for texture analysis, especially in biomedical images [20]. The extracted textured features, including contrast (CON), correlation (CORR), dissimilarity (DISS), angular second moment (ASM), entropy (ENT), and finally, the inverse different moment (IDM) of each feature, were extracted in 0°, 45°, 90°, and 135° directions [21]. The 36 colored features were extracted from the color-converted, enhanced image via a color moment approach. Color moment is a simple feature extraction technique with four features: mean (MEAN), standard deviation (STD), entropy (ENT), and skewness (SKE) extracted from each of the nine color channels [22].

#### 3.4.2. Automatic Feature Extraction

A deep learning structure is an artificial neural network with unbounded number of layers [23]. In a deep architecture, low-level layers extract simple attributes from the raw input, where higher-level layers identify more complex features. Recurrent neural networks (RNN) and convolutional neural networks (CNN) are the most prominent deep learning (DL) algorithms. In this paper, ResNet101 was exploited to extract 1000 features automatically [24,25].

### 3.5. Feature Selection

Feature selection is the process of choosing the most important features that contribute to model learning [26] Most feature selection methods are wrapper methods, which evaluate the features using the learning algorithm. Algorithms based on the filter model examine the intrinsic properties of the data to evaluate the features before the learning tasks. Filter-based approaches almost always rely on class labels, commonly assessing correlations between features and class label [27]. Some typical filter methods include data variance, Pearson correlation coefficients, Fisher score, and the Kolmogorov–Smirnov test. Ensemble based feature selection methods are designed to generate an optimum subset of features by combining multiple feature selectors based on the 20 intuitions behind the ensemble learning. The general idea of ensemble feature selection is to aggregate the decisions of diverse feature selection algorithms to improve representation ability.

This work analyzed other feature selection methods such as Relief (Kira and Rendell) [13], infinite latent feature selection (ILFS), and [28] principal component analysis (PCA) [29,30]. Infinite latent feature selection (ILFS) technique consists of three main steps. The first one is the preprocessing step, then weighting the graph, and the last one is ranking. The goal of the pre-processing stage is to quantify the distribution of features *x_i_* in the matrix format. Then, calculate the value for a specific token so that each feature *x_i_* can be represented by the token *t*; this process is called discriminative quantization [12]. The Fisher criterion method is used to calculate vectors from a feature. The next step is graph weighting. The purpose of the weighting process is to create a fully connected graph in each node that is connecting each feature with the other features [13].

### 3.6. Machine Learning Models

#### 3.6.1. ResNet101

**Figure 6:**
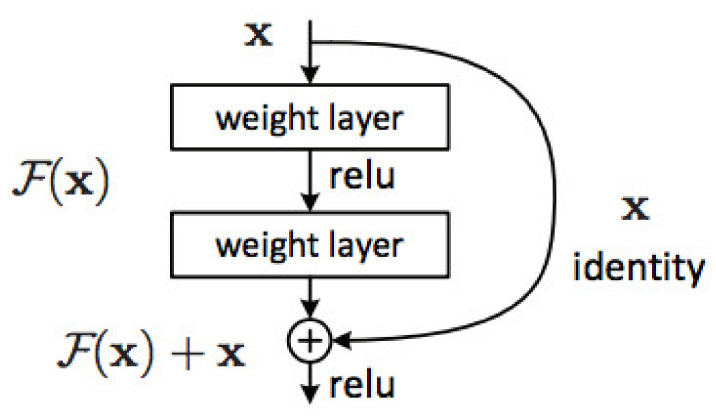
Residual learning: a building block [31].

ResNet101, short for residual networks, is a 101-tier architecture designed by researchers at Microsoft that won the 2015 ILSVRC classification challenge with a 3.57% error rate. It is a neural network that stacks residual blocks with skip connections to solve computer vision and image recognition tasks as shown in Figure 6 [31]. The backbone of the ResNet101 is a convolutional neural network trained on more than one million images from the ImageNet database. As a result, the network learned complex feature representations for a wide range of images and is capable of distinguishing 1000 classes with high performance [32]. Before ResNet101, shallower networks performance was better than that of deeper networks. In other words, increasing the number of layers did not necessarily improve performance; instead, it led to an increase in the training and testing errors because of the exploding gradient problem [33]. ResNet101 allowed scholars to train extremely deep networks without negatively affecting accuracy and performance.

This paper used the pertained ResNet101 model already implemented in MATLAB^®^ version 2021. It consists of 101 layers, and Table 5 details the structure of the used model.

The pretrained CNN utilized the following hyper parameters: the root mean square propagation (RMSProp) optimizer, a patch size of 32, fifteen epochs, and 1 × 10^−4^ learning rate.

#### 3.6.2. Principal Component Analysis (PCA)

Developed in 1933, principal component analysis (PCA) is a widely used data mining technique that can be employed to reveal hidden trends within the data. PCA can help simplify the problem by dramatically reducing the number of features [29,30]. It calculates the principal vectors and uses them to change the basis of the data in an attempt to uncover the concealed truth. The scree plot ranks the vectors according to their signal content, and only the fundamental components that preserve most of the signal information are considered, while the rest are ignored. Smaller datasets are simple and easier to explore and visualize. Therefore, PCA transforms a large group of variables into a smaller set without compromising accuracy. To summarize, dimensionality reduction techniques can help solve problems fast with an acceptable accuracy, and they require less computing power [12,34].

#### 3.6.3. Support Vector Machine (SVM)

A support vector machine (SVM) is a supervised machine learning algorithm developed by Vladimir N. Vapnik in 1963 and refined in the 1990s. It is a binary linear classifier that can be cascaded to solve a multiclass problem. It attempts to find a decision boundary, the maximum marginal hyperplane (MMH), that maximizes the separation region between two categories. SVM converts a linear non-separable classification problem into a separable one by utilizing the kernel trick that transforms a low-dimensional space into a higher-dimensional space. This can be obtained by mapping the used features into a higher-dimensional space using kernel functions such as linear, polynomial, and radial basis function (RBF). In general, kernel selection is based on the type of the transformation and the type of the data [35,36,37]. In this paper, a cascaded SVM classifier that uses Gaussian kernel function is employed in general pattern classification. To improve the performance of the SVM classifier, the dataset is divided into two main subsets: the first one is point-like, and the other one is flaky corneal ulcers, which can be divided further into point-flaky mixed and flaky corneal ulcers. Other image categories (type and grade grading) were kept as is.

**Figure 7:**
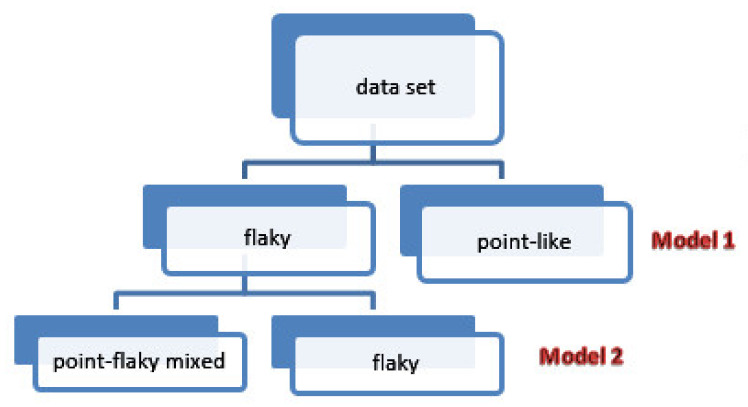
SVM models for general pattern classification.

Figure 7 illustrates the structure of the two models; Model 1 uses an SVM to discriminate between point-like and flaky corneal ulcers. On the other hand, Model 2 is in charge of classifying flaky corneal ulcers further into two other classes, namely flaky-point and point-flaky mixed, using another cascaded SVM classifier.

For grade grading and type grading categories, the multi class SVM is employed to classify grade grading into four classes, whereas type grading is classified into five classes as well. The kernel that has been used is polynomial kernel with order 3.

## 4. Results

**Figure 8:**
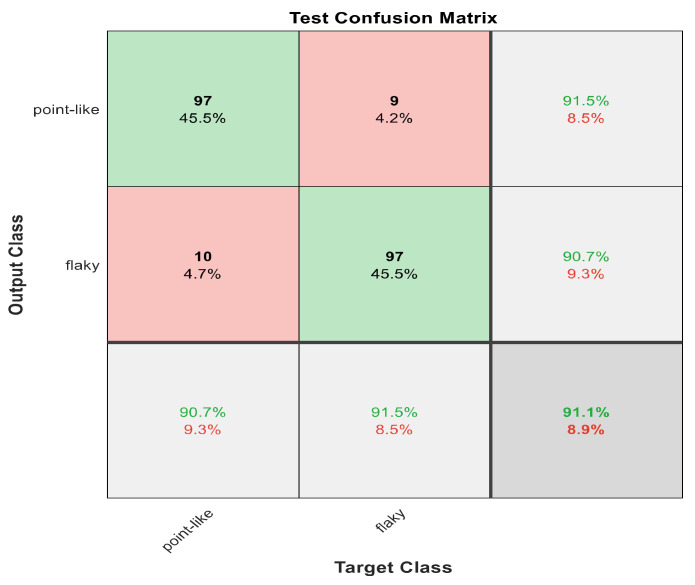
The confusion matrix with ECFS-reduced features for Model 1.

**Figure 9:**
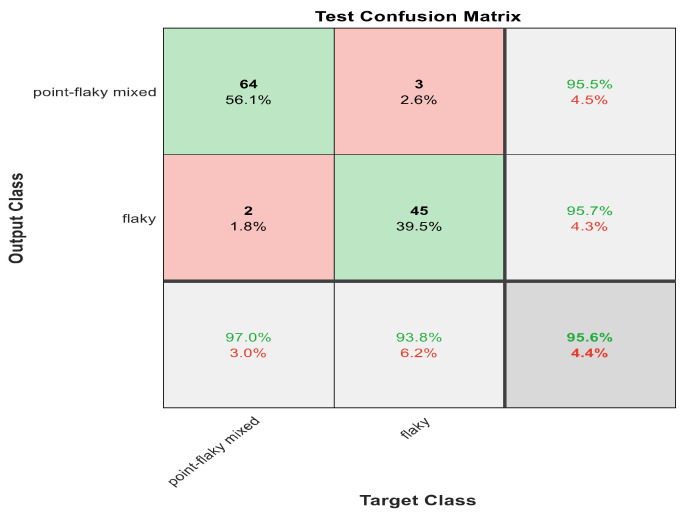
The confusion matrix with ECFS-reduced features for Model 2.

**Figure 10:**
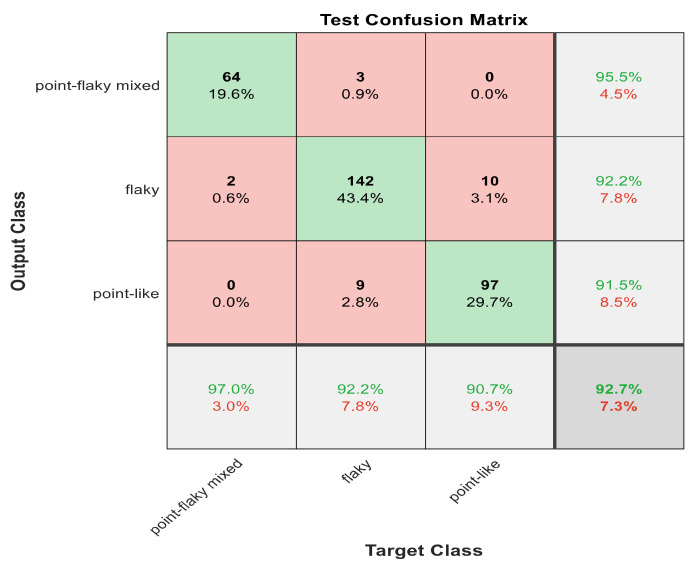
The confusion matrix with ECFS-reduced features for the whole cascading system.

**Figure 11:**
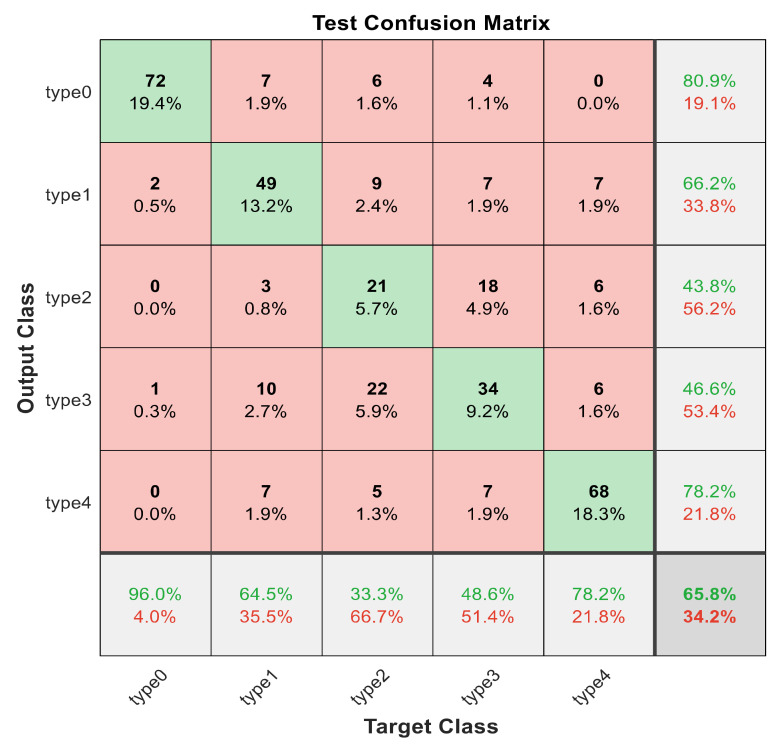
The confusion matrix with ECFS-reduced features for type grading.

**Figure 12:**
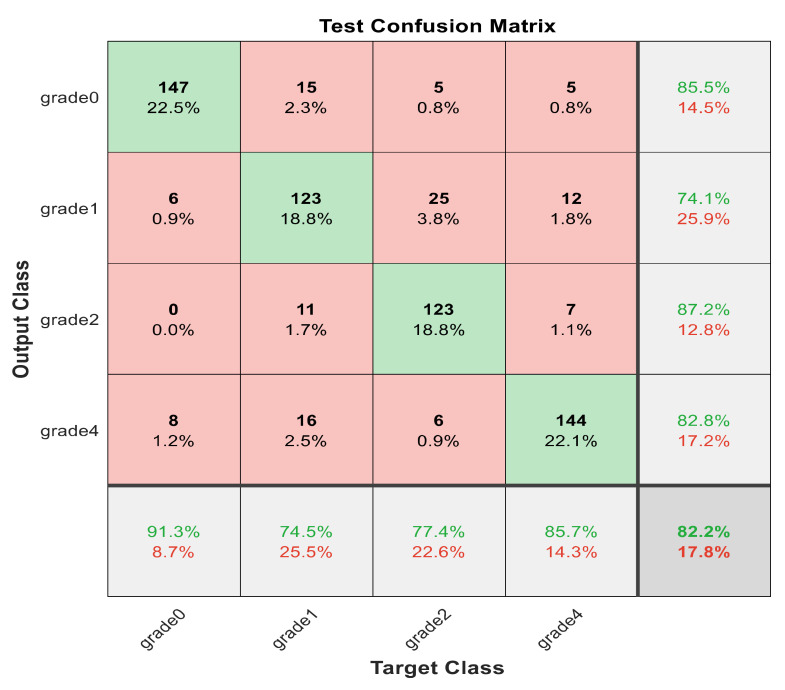
The confusion matrix with PCA-reduced features for grade grading.

**Figure 13:**
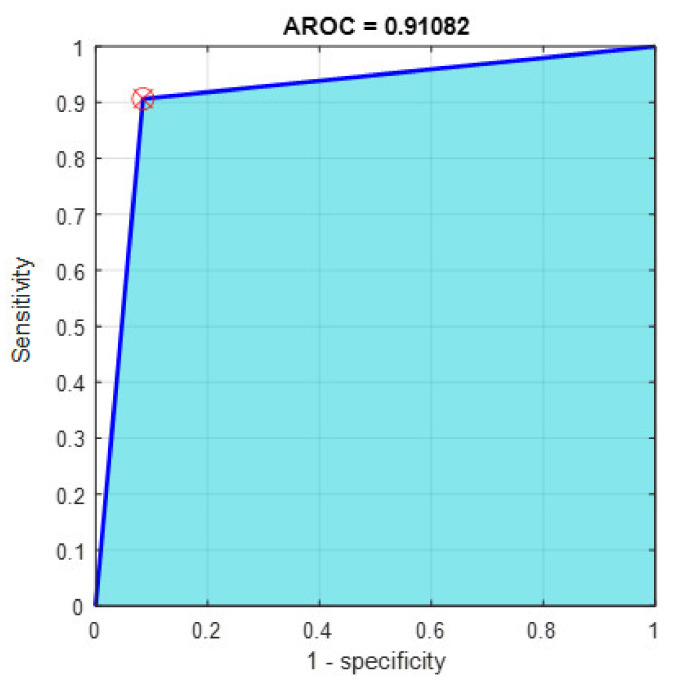
The AROC with ECFS-reduced features for Model 1.

**Figure 14:**
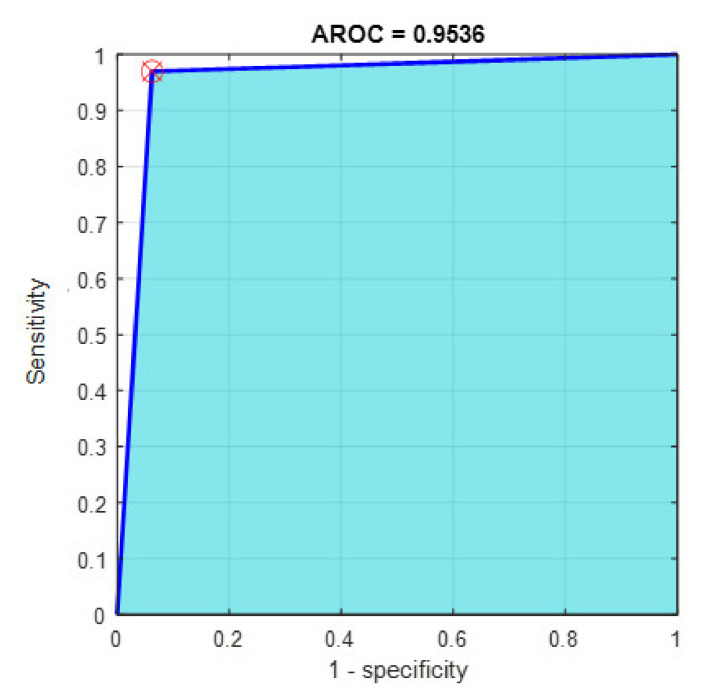
The AROC with ECFS-reduced features for Model 2.

**Figure 15:**
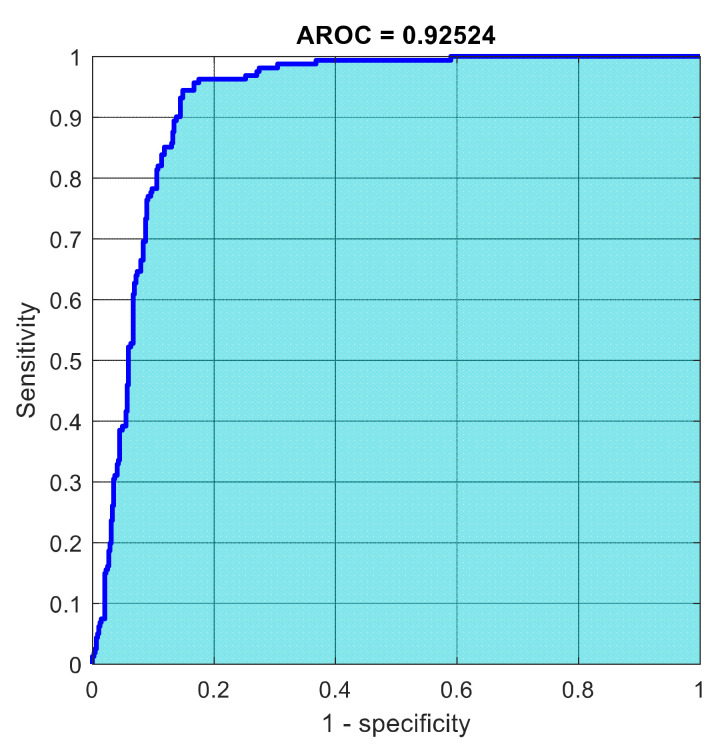
The AROC with ECFS-reduced features for the whole cascading system.

**Figure 16:**
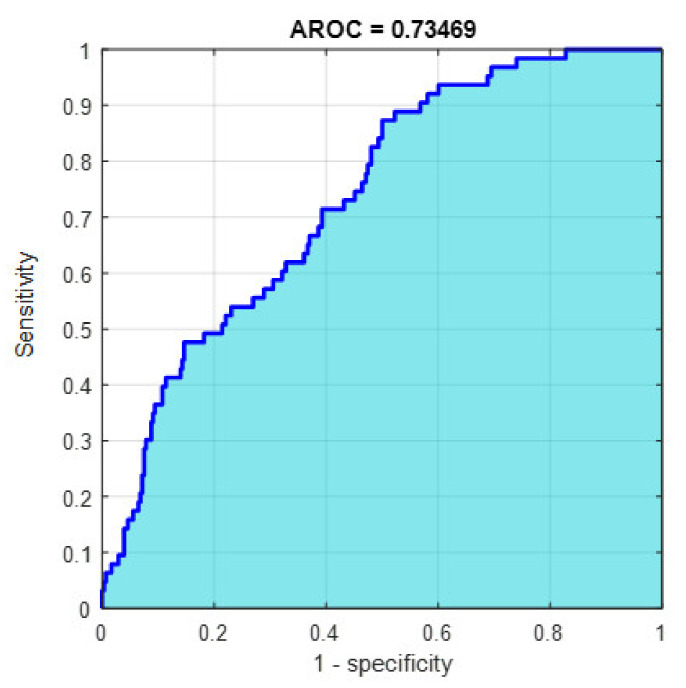
The AROC with ECFS-reduced features for type grading.

This section discusses the detailed results using manual and automatic feature extraction for the general pattern, type grading, and grade grading classification problems. Figure 8, Figure 9, Figure 10, Figure 11 and Figure 12 show the multiclass confusion matrix for all examined models, and the rows represent the predicted category, while the columns represent the real category. It is clear from the figures that all models successfully isolated the concealed features that are associated with each class group. On the other hand, Figure 13, Figure 14, Figure 15 and Figure 16 show the ROC curve for all tested models, and the *x*-axis describes the false-positive rate (specificity), while the *y*-axis represents the true-positive rate (sensitivity).

### 4.1. Manual Feature Extraction

The results in Table 6 show that 30 handcrafted features extracted using ECFS seem to generate better class separability than 60 hand-selected features. Different feature selection methods were employed in the classification process to discover the 30 most significant features. Table 6 also shows that Model 1 using ECFS succeeded in discriminating between flaky and point-like ulcers with 91.1% accuracy, while Figure 8 shows the corresponding confusion matrix. Model 2 using ECFS distinguishes between point-flaky mixed and flaky with an accuracy of 95.6%, and Figure 9 shows the corresponding confusion matrix. Figure 10 shows the confusion matrix for the whole cascading system with features selection ECFS.

Figure 8, Figure 9, Figure 10, Figure 11 and Figure 12 describe the confusion matrix for all classification problems using 30 most significant features extracted with ECFS. Figure 8 shows the confusion matrix for the first model, a maximum accuracy of 91.1% was reached for discriminating flaky and point-like ulcers. Figure 9 shows the confusion matrix for the second model, and a maximum accuracy of 95.6% was reached for distinguishing between flaky and point-flaky mixed ulcers. Figure 10 clarifies the overall accuracy for the cascading classifier. Its maximum accuracy is 92.2%. For type grading, the model managed to discriminate between all five types with a 65.8% accuracy as shown in Figure 11. For grade grading, the model succeeded in distinguishing between all four grades with an 82.2% accuracy as shown in Figure 12. Table 6 summarizes the results for different classification problems using different features reduction methods (PCA, ECFS, ILFS, and Fisher).

**Figure 17:**
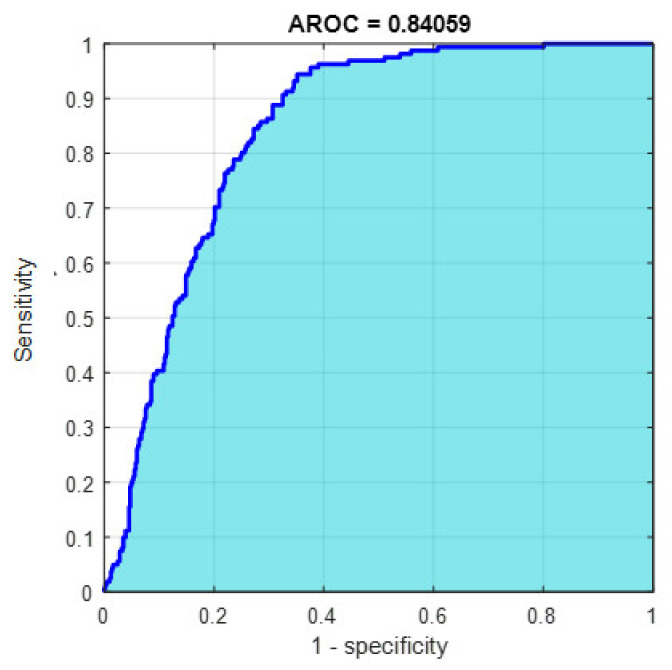
The AROC with PCA-reduced features for grade grading.

However, Figure 13, Figure 14, Figure 15, Figure 16 and Figure 17 show the receiver operating characteristics curve (ROC) for each confusion matrix that has been mentioned above, respectively. Each one describes the specificity and sensitivity for each classifier beside the area of the receiver operating characteristic curve (AROC).

### 4.2. Automatic Feature Extraction

**Figure 18:**
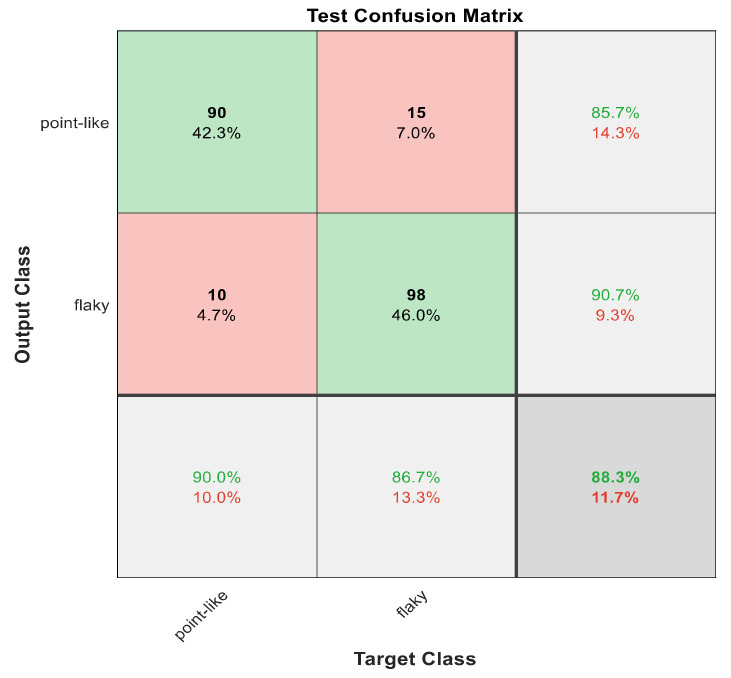
The confusion matrix for Model 1 using 1000 deep learning features.

**Figure 19:**
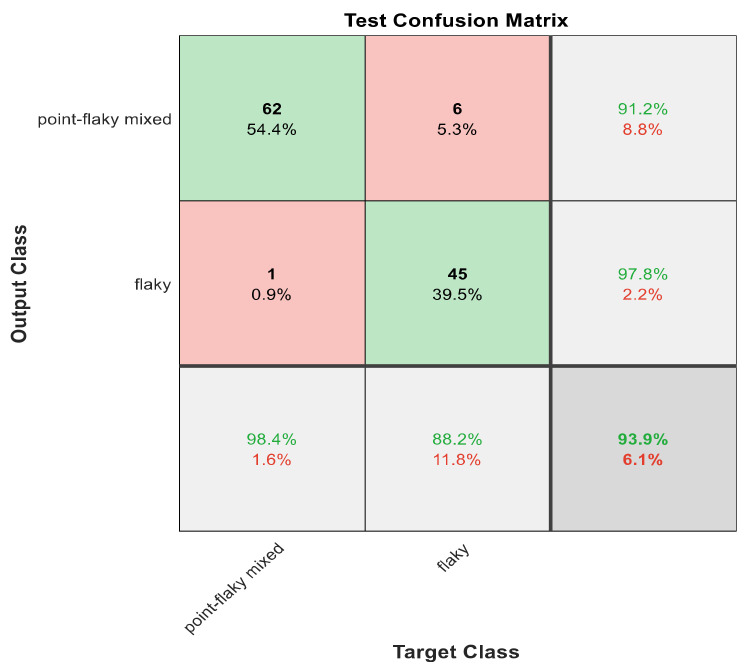
The confusion matrix for Model 2 using 1000 deep learning features.

**Figure 20:**
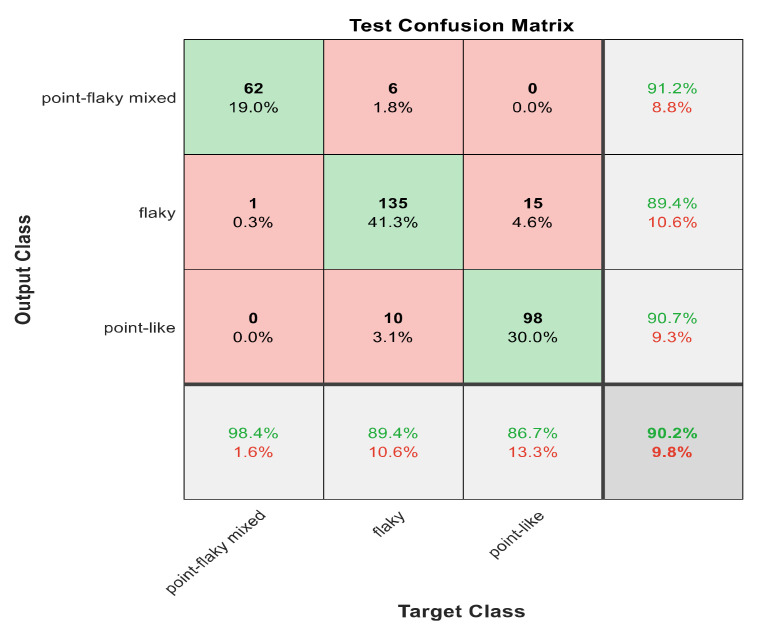
The confusion matrix for whole cascading system using 1000 deep learning features.

**Figure 21:**
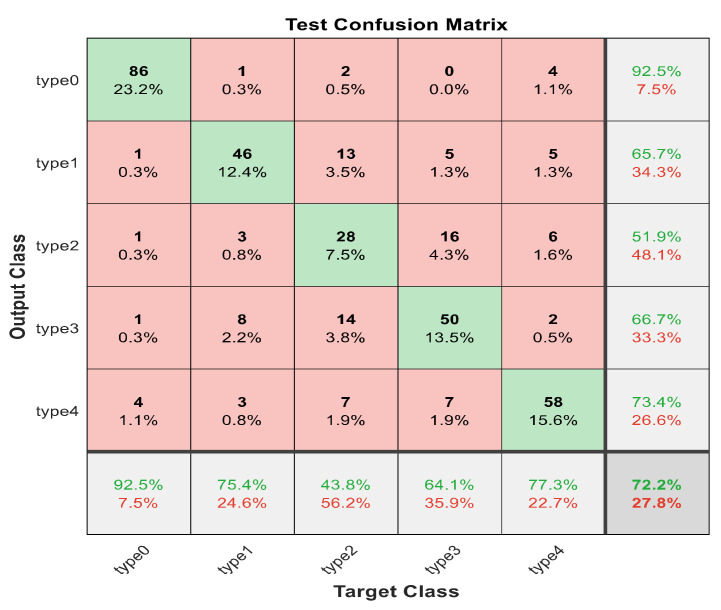
The confusion matrix with for type grading using 1000 deep learning features.

**Figure 22:**
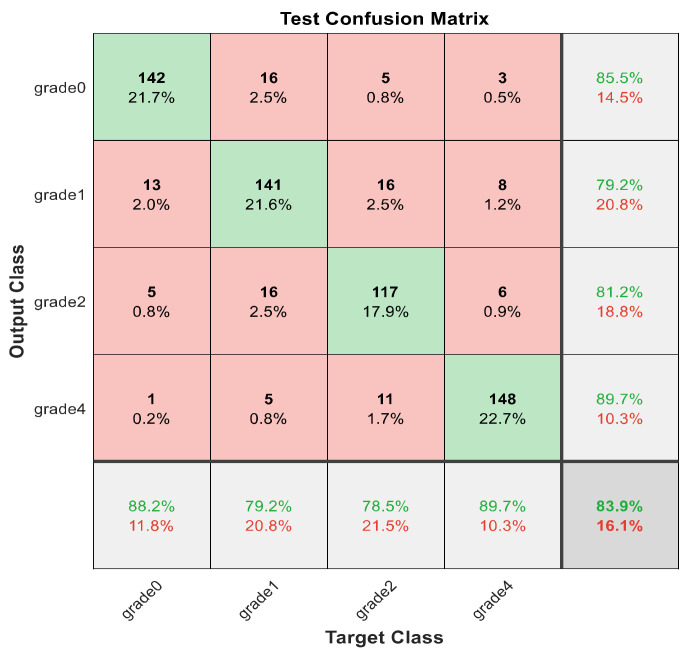
The confusion matrix with for grade grading using 1000 deep learning features.

One thousand features were automatically extracted using the ResNet101 pretrained model; applying different dimensionality reduction techniques reduced the number of features from 1000 to 30 and 50 features. Figure 18, Figure 19, Figure 20, Figure 21 and Figure 22 describe the confusion matrix for all classification problems using 1000 automatically extracted features. Figure 18 shows the confusion matrix for the first model, and a maximum accuracy of 88.3% was reached for discriminating flaky and point-like ulcers. Figure 19 shows the confusion matrix for the second model, and a maximum accuracy of 93.9% was reached for distinguishing between flaky and point-flaky mixed ulcers. For type grading, the model managed to discriminate between all five types with a 72.2% accuracy as shown in Figure 21. Furthermore, the overall accuracy for whole cascading system for three classes is presented in Figure 19; it reaches to 90.2%. For grade grading, the model succeeded in distinguishing between all four grades with an 83.9% accuracy as shown in Figure 22. Table 7 and Table 8 summarize the results for all classification problems using 30 and 50 features, respectively.

The results in Table 7 and Table 8 show that 1000 automatically extracted features seem to generate better class separability than 30 or 50 selected features. Table 7 and Table 8 also show that Model 1 using 1000 features succeeded in discriminating between flaky and point-like ulcers with 88.3% accuracy, while Figure 18 shows the corresponding confusion matrix. Model 2 using 1000 features distinguished between point-flaky mixed and flaky with an accuracy of 93.9%, and Figure 19 shows the corresponding confusion matrix. On the other hand, the overall accuracy of the cascading system reaches a maximum accuracy of 90.2%, and Figure 20 describes its result.

**Figure 23:**
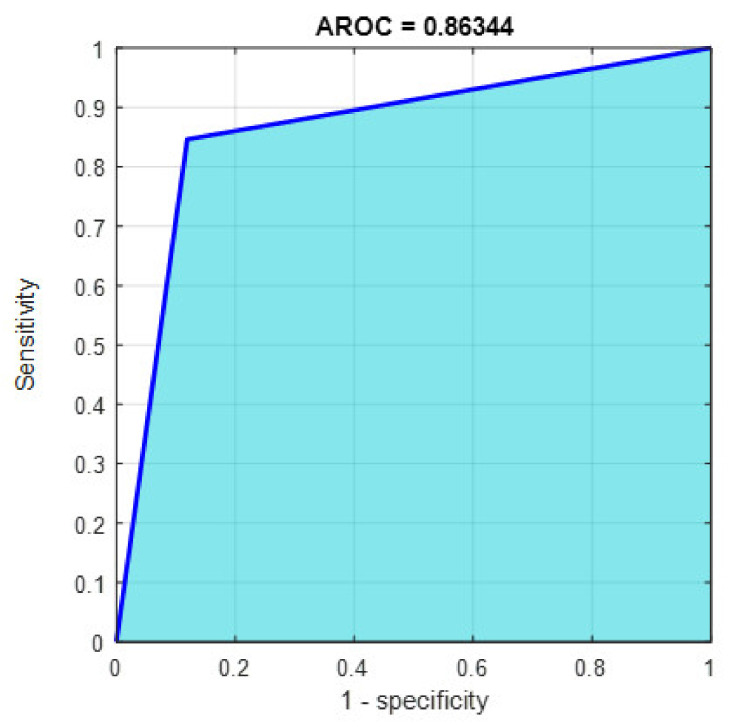
The AROC for Model 1 using 1000 deep learning features.

**Figure 24:**
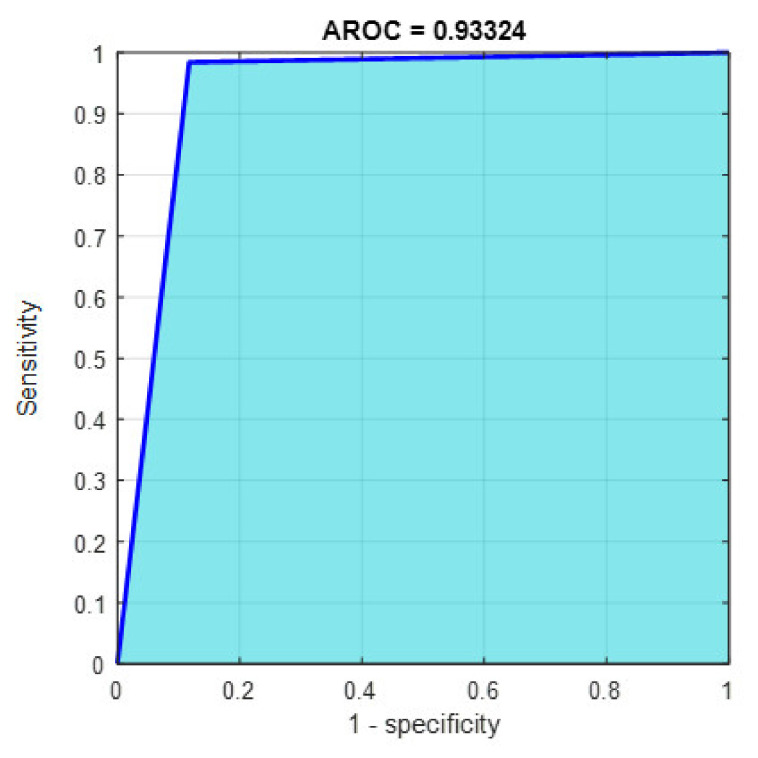
The AROC for Model 2 using 1000 deep learning features.

**Figure 25:**
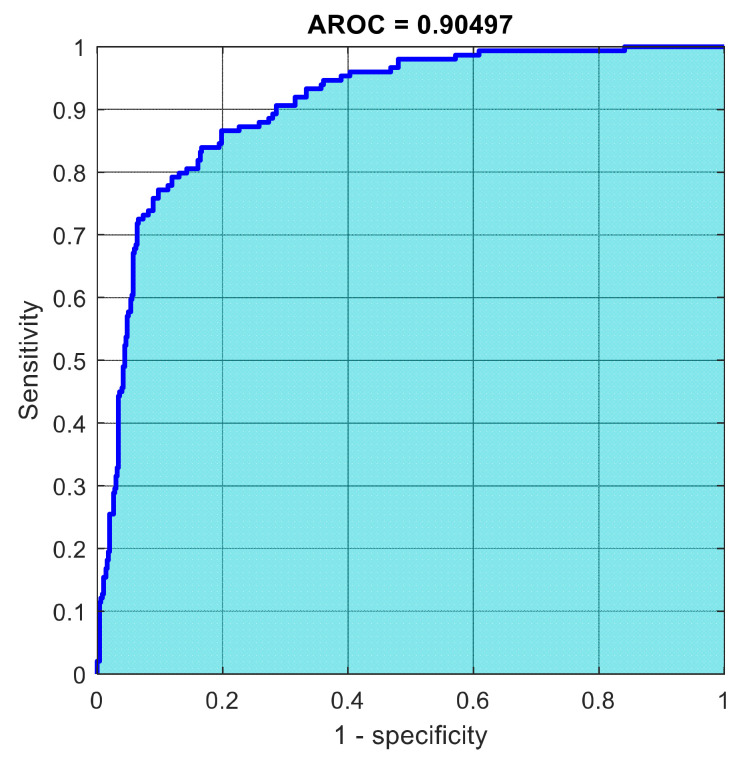
The AROC for cascading model using 1000 deep learning features.

**Figure 26:**
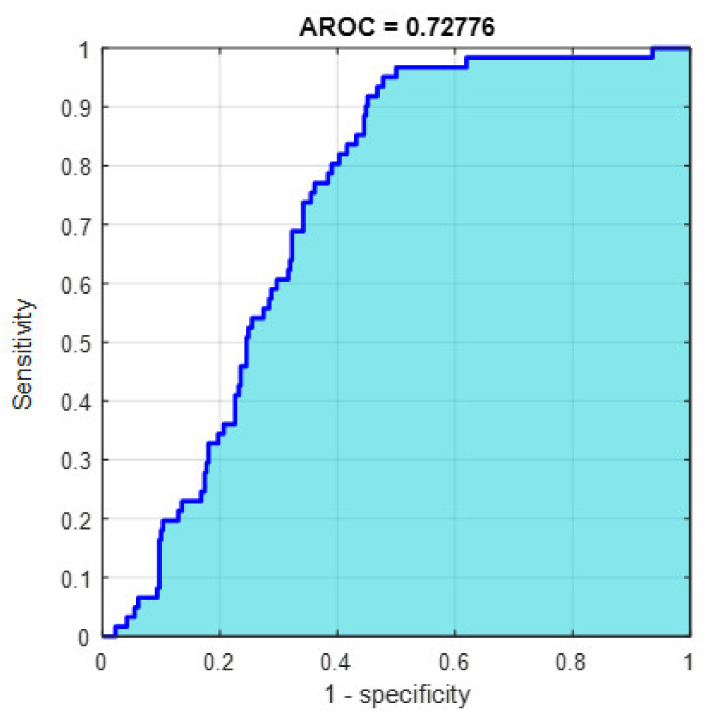
The AROC for type grading using 1000 deep learning features.

**Figure 27:**
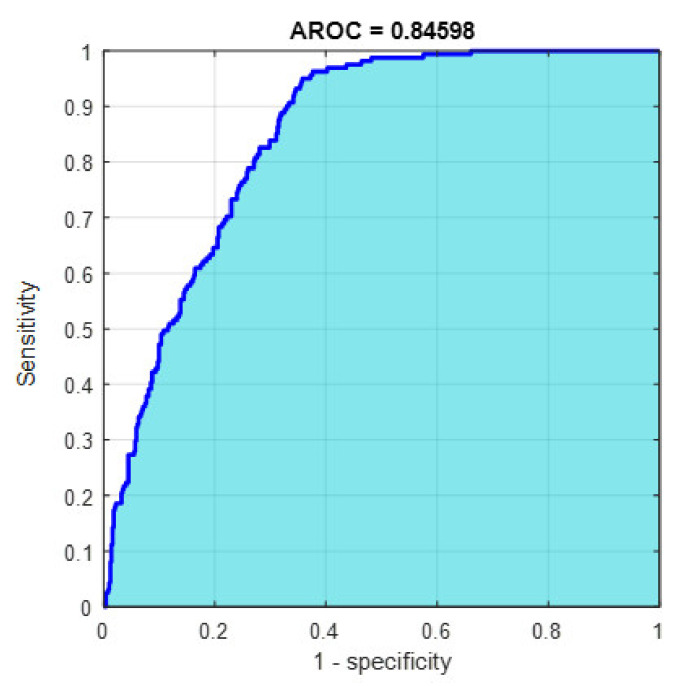
The AROC for grade grading using 1000 deep learning features.

Figure 23, Figure 24, Figure 25, Figure 26 and Figure 27 show the AROC curve for each of the above confusion matrix.

## 5. Discussion

Table 6 shows that the results of the 30 most significant hand-crafted features (with selection) are at least 5% better than those attained using 60 features (without selection). Selecting features by hand extracts both related and unrelated attributes, while applying various reduction techniques extracts the most significant features and eliminates noisy unrelated features. The best results were obtained using the ECFS reduction technique, but for the grade grading problem, PCA outperformed ECFS. One thousand features were automatically extracted using the ResNet101 deep learning model, and the most significant 30 and 50 features were obtained utilizing different dimensionality reduction techniques. It is clear from Table 7 and Table 8 that the results attained using the 50 most significant features are better than those obtained utilizing 30 most important features. In addition, the results attained using 1000 features (without selection) are better than those obtained utilizing feature reduction (30 and 50). Training using a higher number of features (nearly 200x) includes more signal content and results in improved performance. Utilizing the 50 most important features, PCA was the best reduction technique, and it gave comparable (nearly 2% off) performance to those attained using all 1000 features.

**Figure 28:**
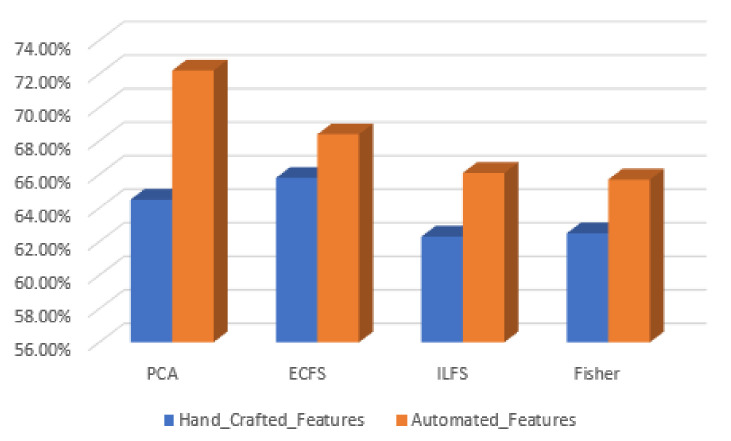
Accuracy for the 30 most significant features for type grading in both automated and hand-crafted features.

**Figure 29:**
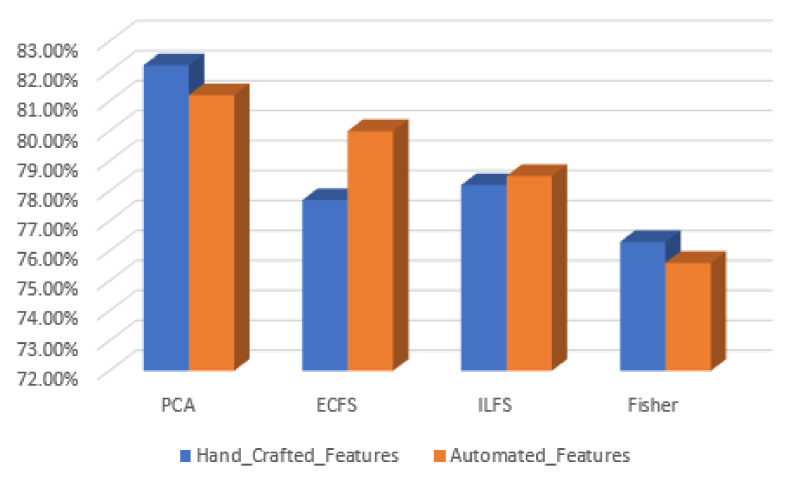
Accuracy for the 30 most significant features for severity grading in both automated and hand-crafted features.

In discriminating between various classes of type grading, the performance of the most significant 30 automated features are better than the 30 most important features by manual feature extraction. This is clear in Figure 28, where the PCA gives the highest accuracy, reaching 72%. However, the behavior of hand-crafted features for the 50 most significant features is better than deep learning descriptors for distinguishing different severity grading classes. The best feature reduction techniques in this scenario are achieved by PCA, as is clear in Figure 29.

**Figure 30:**
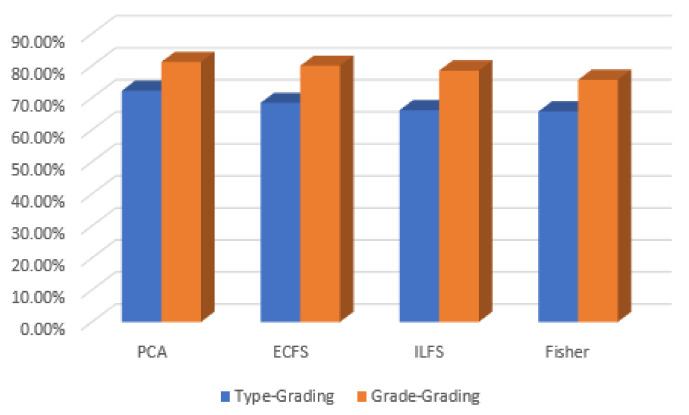
Accuracy for the 30 most significant features for severity grading and type grading employing automatic features.

**Figure 31:**
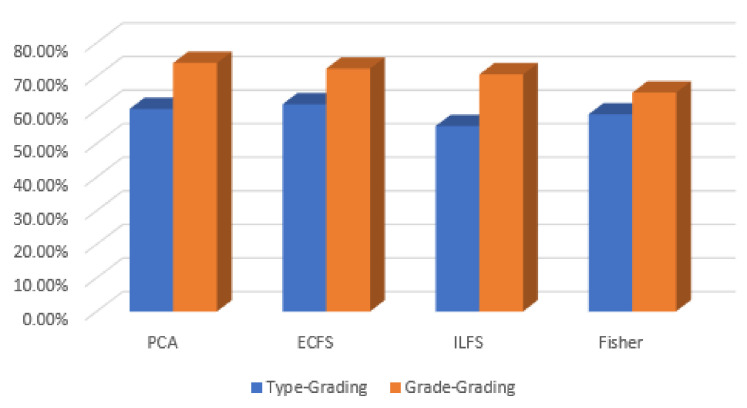
Accuracy for the 50 most significant features for severity grading and type grading employing automatic features.

Table 6, Table 7 and Table 8 show that the results attained using features extracted automatically are better than those extracted manually for type/grade grading classification problems. Those are complex problems that utilizes multiclass classifiers; the higher the number of classes to categorize, the higher the number of features needed to successfully perform the task, which is also clear in Figure 30 and Figure 31. Table 6, Table 7 and Table 8 also show that extracting features manually is better than extracting them automatically for binary classification problems (Models 1 and 2). Those are simple problems that utilize cascaded binary classifiers; the lower the number of classes, the lower the number of features needed to successfully perform the task. In addition, features extracted automatically are higher in number, and they add more noise and might cause overfitting. In general, those mixed results can be explained by the fact that features extracted automatically are higher in quantity but not necessarily better in quality, while not all automatically extracted features correlate with different class categories.

Differences and commonalities between this study and other studies are that this is the first report—based on our knowledge—to explore the classification of the corneal ulcers in both the grade grading and type grading categories. Although the poor quality of the images used in this study resembles real-life challenges, building a robust model needs larger, more diverse, and higher-quality images, and this is a limitation of this study. Moreover, treatment for corneal ulcers varies depending on the cause of ulcer. Corneal ulcers can occur due to bacterial infection, viral infection, amoeba infection, or inflammatory response. As a result, differential diagnosis is very important, for the treatment is different. This research lacked consideration of this aspect, and this is considered another limitation. Finally, the presented results can help build a robust and reliable deep learning-based model that can assist doctors in rural areas or primary care units in performing clinical diagnosis of keratitis early, correctly, and automatically.

## 6. Conclusions

Early and timely detection of corneal ulcers is crucial for preventing the progression of the infection. This study presented an automated classification method for distinguishing different corneal ulcer patterns, including general pattern, specific pattern (type grading), and severity degree (grade grading). Data were augmented, and several image processing techniques were applied, including morphological opening, adjusting, and histogram equalization to improve the performance of the suggested approach. The study also attempted to obtain a comparable performance using a smaller number of features by applying various feature selection methods, such as ILFS, ECFS, Fisher, and PCA. The proposed system used the SVM classifier to discriminate between different ulcer patterns.

While classifying type grading, the automated features performance was better than that of the hand-crafted approach using the 30 most important features, whereas in classifying grade grading, the results were mixed, and there was no significant difference between automatic and manual feature extraction using the 30 most relevant features. General grading using the cascaded system achieved the best results either using automated features or manual features. We attribute this result to the use of cascading SVM, which is responsible for finding the most significant features in each stage and obtaining higher accuracy and higher sensitivity for all three classes. The performance of the deep learning model in classifying severity grading was better than classifying type grading using various reduction techniques. This result holds regardless of the number of features used, whether 50 or 30 features.

## Figures and Tables

**Table 1:** The distribution of the images used in the system.

General Pattern	Point-Like	Point-Flaky Mixed	Flaky		
Number of images	358	263	91		
**Type grading (specific pattern)**	**Type 0**	**Type 1**	**Type 2**	**Type 3**	**Type 4**
Number of images	36	98	203	273	102
**Grade grading (severity degree)**	**Grade 0**	**Grade 1**	**Grade 2**	**Grade 3**	**Grade 4**
Number of images	36	78	50	50	548

**Table 2:** Augmentation Process for General Grading Dataset.

General Grading Class	Point Like	Flaky	Point-Flaky Mixed
Before Augmentation	358	91	263
After Augmentation	358	182	263
Augmentation Multiplier	0	2	0

**Table 3:** Augmentation Process for Type Grading Classes.

Type Grading Classes	Type 0	Type 1	Type 2	Type 3	Type 4
Before Augmentation	36	98	203	273	102
After Augmentation	288	294	203	273	306
Augmentation Multiplier	8	3	0	0	3

**Table 4:** Augmentation Process for Grade Grading Classes.

Grade Grading Classes	Grade 0	Grade 1	Grade 2 and 3	Grade 4
Before Augmentation	36	78	50	548
After Augmentation	540	624	550	548
Augmentation Multiplier	15	8	11	0

**Table 5:** The stricter of ResNet101 [31].

Layer Name	Output Size	ResNet101
**Conv1**	112 × 112	7 × 7, 64, stride 2
**Conv2_x**	56 × 56	3 × 3 max pool, stride 2
		$\left[\begin{array}{c} 1\times 1,\;64\\ 3\times 3,\;64\\ 1\times 1,\;256 \end{array}\right]\times 3$
**Conv3_x**	28 × 28	$\left[\begin{array}{c} 1\times 1,\;128\\ 3\times 3,\;128\\ 1\times 1,\;512 \end{array}\right]\times 4$
**Conv4_x**	14 × 14	$\left[\begin{array}{c} 1\times 1,\;256\\ 3\times 3,\;256\\ 1\times 1,\;1024 \end{array}\right]\times 23$
**Conv5_x**	7 × 7	$\left[\begin{array}{c} 1\times 1,\;512\\ 3\times 3,\;512\\ 1\times 1,\;2048 \end{array}\right]\times 3$
	1 × 1	Average pool, 1000-d FC, softmax

**Table 6:** Testing accuracy results using 30 most significant hand-crafted features.

Image Categorization	General Pattern		Type Grading	Grade Grading
	Model 1	Model 2		
60 features	84.5%	89%	60.9%	74.5%
PCA	85.2%	94%	64.5%	**82.2%**
ECFS	**91.1%**	**95.6%**	**65.8%**	77.7%
ILFS	88%	90%	62.3%	78.2%
Fisher	87%	93.6%	62.5%	76.3%

PCA, principal component analysis; ECFS, ensemble-based classifier feature selection; ILFS, infinite latent feature selection.

**Table 7:** Testing accuracy results using 30 most significant automatic features.

Image Categorization	General Pattern		Type Grading	Grade Grading
	Model 1	Model 2		
1000 features	**88.3%**	**93.9%**	**72.2%**	**83.9%**
PCA	72.3%	80.7%	60.3%	74.0%
ECFS	69.1%	75.1%	61.7%	72.3%
ILFS	69.5%	79.3%	55.2%	70.6%
Fisher	65.2%	72.9%	58.7%	65.2%

PCA, principal component analysis; ECFS, ensemble-based classifier feature selection; ILFS, infinite latent feature selection.

**Table 8:** Testing accuracy results using 50 most significant automatic features.

Image Categorization	General Pattern		Type Grading	Grade Grading
	Model 1	Model 2		
1000 features	**88.3%**	**93.9%**	**72.2%**	**83.9%**
PCA	86.4%	91.2%	72.2%	81.2%
ECFS	75.9%	86.5%	68.4%	80.0%
ILFS	70.6%	79.3%	66.1%	78.5%
Fisher	74.6%	84.2%	65.7%	75.6%

PCA, principal component analysis; ECFS, ensemble-based classifier feature selection; ILFS, infinite latent feature selection.

## Data Availability

The dataset analyzed during the current study was derived from the following public domain resources SUSTech-SYSU dataset. Available online: https://github.com/CRazorback/The-SUSTech-SYSU-dataset-for-automatically-segmenting-and-classifying-corneal-ulcers (1 February 2022).
